# How to set the agenda for hepatitis C: a theory-driven policy analysis

**DOI:** 10.1186/s12961-022-00824-3

**Published:** 2022-02-14

**Authors:** Julia Kind, Bettina Maeschli, Philip Bruggmann

**Affiliations:** 1 Arud Centre for Addiction Medicine, Schützengasse 31, 8001 Zurich, Switzerland; 2 Swiss Hepatitis, c/o Arud, Schützengasse 31, Zurich, Switzerland; 3 Institute of Primary Care (IHAMZ), University and University Hospital of Zurich, Pestalozzistrasse 24, Zurich, Switzerland

**Keywords:** HCV, Elimination, Multiple streams framework, Qualitative interviews, Policy entrepreneur, Swiss Hepatitis, Window of opportunity, Switzerland, National strategy

## Abstract

**Background:**

Hepatitis C virus (HCV) represents a significant public health burden. When new HCV drugs arrived in 2014, the disease became curable, but the administration remained reluctant to address this public health issue. However, the Swiss parliament recently decided to integrate HCV into the next national HIV programme. This study investigates how HCV came onto the political agenda in Switzerland and which actors and factors were influential in this process.

**Methods:**

The data collection is based on document analysis and semi-structured interviews. The transcripts were coded by deriving the codes from the data in terms of content followed by the application of the multiple streams framework.

**Results:**

Health authorities, unlike experts, did not see the HCV epidemic as a relevant public health threat. Due to cost-related restriction of access to treatment, the potential of the new HCV drugs could not be fully exploited. The administration’s position proved difficult to change, despite evidence to the contrary. For 30 years, authorities set the agenda in health policy regarding HCV, unheeded by politicians. But recently, a policy entrepreneur has for the first time successfully managed to put HCV on the political agenda. After years of education and lobbying, it used the window of opportunity in the form of the new edition of the national HIV programme. The parliamentary decision to include HCV in this programme broke the long-standing primacy of the administration in the field of HCV, which had long prevented a more active handling of the HCV field.

**Conclusions:**

The case of HCV in Switzerland shows that evidence alone is not enough to bring about health policy changes. A policy entrepreneur is needed who overcomes resistance, brings together the three streams—problem, policy and political—and exploits the window of opportunity at the right time. To be successful, the policy entrepreneur must identify the indicators that map the problem, network and convince decision-makers, recognize policy windows and use them—as has been the case with HCV in Switzerland.

## Background

An estimated 71 million people, around 1% of humanity, are infected with the hepatitis C virus (HCV) [1]. If left untreated, the disease may lead to liver cirrhosis, liver cancer and ultimately to death. Although around 400,000 people worldwide die each year as a result of the infection [1], HCV has received little attention for many years as a public health-relevant disease [2]. This only began to change 7 years ago with the arrival of new, highly effective therapies, the direct-acting antivirals (DAAs). Although since 1991 there had already been an initial therapy with interferon (from 2000 with pegylated interferon), supplemented with the active substance ribavirin as a combination therapy in 1998, the cure rates were modest and the side effects severe [3]. Modern DAAs, on the other hand, lead to a cure in over 95% of cases [4], have hardly any side effects and shorten the duration of therapy from 36–48 to 8–12 weeks [5].

WHO saw this breakthrough as an opportunity to set an ambitious target: to eliminate the HCV globally by 2030 [6]. Switzerland has signed the corresponding WHO resolution [7]. However, since there are few new infections in Switzerland today, the administration saw no need for coordinated action for elimination of HCV on the national level. Instead, due to the high cost, the new therapies were initially approved only for people with advanced liver damage. This stood in the way of rapid elimination. Only in 2017, 3 years after approval, was this restriction lifted. Even so, major hurdles remain to be overcome to achieve elimination: of the estimated 40,000 people with hepatitis C in Switzerland, just around 26,000 are aware of their infection, and only 1100 people currently receive any treatment at all each year [8]. Thus, despite treatment availability, 200 people in Switzerland die each year as a result of an HCV infection, five times more than of HIV [9]. These figures demonstrate why there is talk of a silent epidemic [10] in connection with HCV: there is a lack of awareness of the disease among the public, in politics and administration, but also among health professionals.

Thanks to the new drugs, the situation has changed significantly since 2014, both from a medical and a public health perspective. New actors have emerged, scientific knowledge has evolved, and in principle, the disease could be eliminated. Against this backdrop, our study uses Kingdon’s multiple streams framework (MSF) [11] to show which actors are involved in the policy field of hepatitis C and how the different actors have influenced its formulation, with a focus on developments since the introduction of the new DAA drugs.

## Methods

The data collection was based on the analysis of documents and interviews. The documents analysed include reports, scientific publications, newspaper articles, press releases, parliamentary interventions and official regulations. From the analysis, the processes were reconstructed and the various events traced. The document analysis then led to the identification of key actors and decision-makers, who were asked to be interviewed. A total of 11 people were interviewed in open, semi-structured interviews conducted with the help of interview guidelines.

The data analysis took place in two ways: for the material reconstruction [12], the findings from the document analysis, from relevant secondary literature and from the evaluated interviews were combined. On the other hand, the interviews, digitally recorded and subsequently transcribed, were evaluated using a thematic content analysis [13]. For the analysis, the data was processed using ATLAS.ti software. For this purpose, the transcripts were coded by deriving the codes from the data in terms of content. Finally, the MSF [11] was applied in the second part of the analysis [14]. In this process, the codes were revised and combined where necessary, to create higher-level codes.

### Theoretical framework

Kingdon's MSF (MSF) [11] helps to explain why certain issues appear on the political agenda while others do not. It does not assume a linear and rational agenda-setting process where problems are identified and solutions sought, but rather that events occur simultaneously and largely independently of each other. The MSF thus differentiates between three so-called streams, which, when they coincide, create a policy window for the issue to appear on the political agenda. A policy entrepreneur plays a decisive role and is central in three phases: in the selection of those indicators that best reflect the problem; in the process of raising the awareness of decision-makers and the public, sensitizing them to the issue; and in the identification of a policy window and the coupling of the three streams. Without such an actor to connect the three streams, there is a high risk that the opportunity will be lost and the streams will continue to flow independently [11].

In the problem stream, pressing issues come to the attention of people in government circles, requiring either focusing events (e.g. financial collapse) or an indicator (e.g. an alarming statistic). Indicators are particularly important because “constructing an indicator and getting others to agree to its worth become major preoccupations of those pressing for policy change” [11]. Feedback on existing policies or the renewal of a government programme also represent opportunities for a government to take notice of an issue. For assessing issues, Kingdon emphasizes that “conditions become defined as problems when we come to believe that we should do something about them” [11]. In this context, personal values, comparisons and the categorization of an issue are decisive in determining whether a condition is perceived as a problem. Changes in the problem or policy stream can lead to a condition being re-evaluated and then classified as a problem—or vice versa.

The policy stream is the domain of experts, researchers and academics, consultants or administrators, who are active behind the scenes, and are therefore also called “hidden participants”. They develop, discuss and reject ideas and solutions. This is where the foundations for new programmes, draft legislation or even political initiatives are created. To have a chance of survival, ideas and solutions must be (i) technically feasible and easy to implement, (ii) aligned to the values of the specialist community and (iii) practicable at a cost the public and decision-makers find acceptable [11].

The political stream is influenced by factors such as public sentiment, party ideologies, election and voting results, managerial fluctuations in public administration, and activities of interest groups. Changes and shifts taking place in this sphere are powerful agenda-setters. This stream is dominated by the “visible participants” prominent in the media and the public sphere [11].

## Results

### Problem stream

**Fig. 1:**
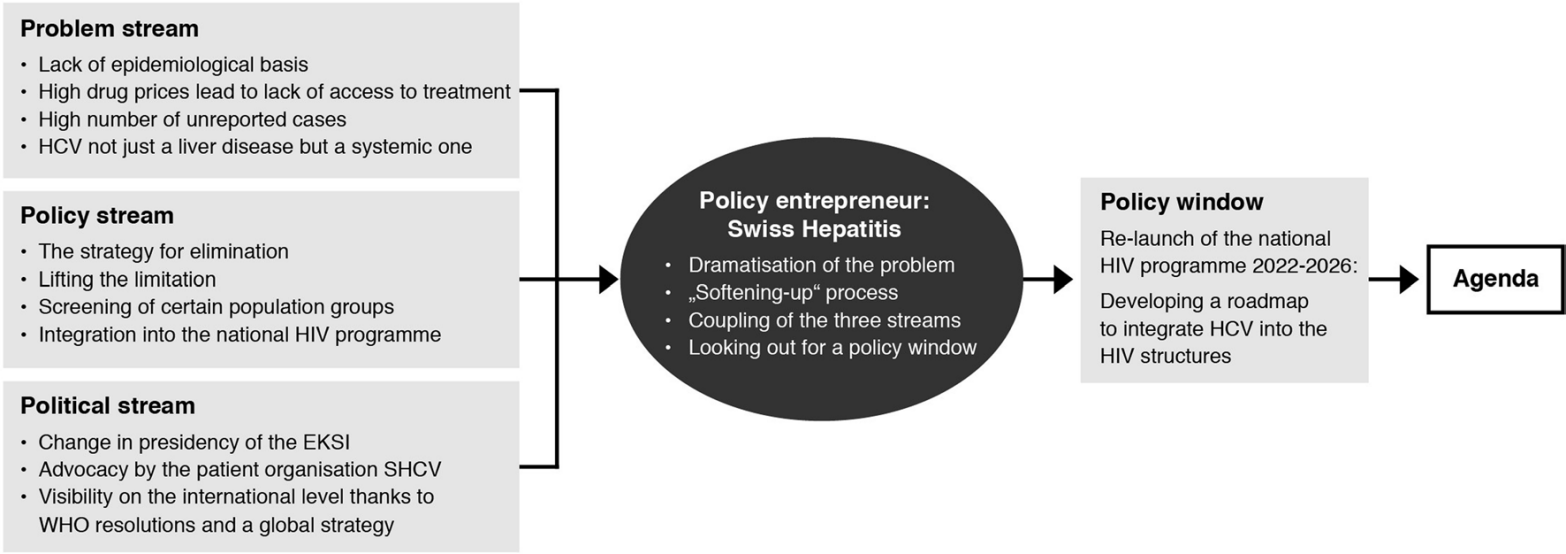
Multiple streams framework by Kingdon applied for hepatitis C policy. The civil society association Swiss Hepatitis acted as a policy entrepreneur by coupling the three streams and seizing the opportunity to set hepatitis C on the political agenda when a policy window opened up with the relaunch of the national HIV programme

In the context of new therapy options with the DAAs and the resulting challenges, for example in respect to the pricing, past failures became particularly prominent. However, not all actors involved agreed in their assessment of the situations mentioned below, in particular whether these represented merely a condition or a problem—that is, whether something had to be done about the situation or not. In fact, there was a marked division between hepatitis experts and representatives of the Federal Office of Public Health (FOPH) (Fig. 1).

#### Lack of epidemiological basis

To date, there is a lack of solid epidemiological data on hepatitis C in Switzerland to provide a robust basis for decision-making about possible measures. The lack of such data became conspicuous in relation to the antiviral drugs with the potential for eliminating hepatitis C. Initially, as a basis for forecasts on treatment with the new drugs, the FOPH assumed that there were about 80,000 HCV-infected people [15, 16]. These inaccurate projections were later corrected to 40,000 [17]. Nevertheless, the original number had led the FOPH to a considerable overestimation of annual treatment figures.

#### High drug prices lead to lack of access to treatment

The initial market price of Sovaldi, the first of the novel DAA drugs, at CHF 84,000 per treatment [18–20], made HCV an economic problem for the authorities. While doctors welcomed the breakthrough of this highly effective therapy, the authorities feared an enormous increase in health insurance premiums [21, 22]. Therefore, they limited the use of the DAAs, considerably more expensive than interferon therapy, to HCV patients with advanced liver damage. This measure, introduced for economic reasons but justified on medical grounds, was only lifted 3 years later [23]. Consequently, only a relatively small number of patients could be treated; patients without advanced liver damage but with other HCV symptoms like fatigue and a higher risk for sequelae such as diabetes and cardiovascular diseases were left behind.

#### High number of unreported cases

The new DAAs and the declared goal of eliminating viral hepatitis in Switzerland by 2030 have highlighted a condition that had long been known but had not been in focus given the limited treatment options with interferon: that many people with an HCV infection were unaware of their infection, or diagnosed but not treated [24].

#### Hepatitis C: not just a liver disease but a systemic one

Growing scientific evidence showed that HCV affects not only the liver, but potentially any organ, and that early therapy can prevent secondary diseases beyond liver damage [25]. This also changed the indication for treatment: the condition of the liver alone as a measure of disease was no longer adequate [26]. Hepatitis C viewed as a systemic disease thereby seriously questioned the FOPH’s limitation: if seen as purely a liver disease, the limitation was reasonable, but consideration of HCV as a systemic, infectious disease demanded treatment independent of the condition of the liver, for as many people as possible.

### Policy stream: the experts’ sphere

The vision of hepatitis elimination serves as a common thread in the policy stream: the individual solutions proposed all aim to make this vision a reality. The various actors, mainly physicians at university hospitals, are well networked and coordinate their activities and research within the framework of Swiss Hepatitis (Fig. 1).

#### The strategy for elimination

When as of 2012 it became clear that new drugs would revolutionize the treatment and even cure HCV, individual experts already began outlining the vision of eliminating viral hepatitis. However, universal approval remained elusive; to some experts the vision seemed too bold and unrealistic, thus being denied “technical feasibility”. Nevertheless, the intensive discussions among the hepatitis experts during this period eventually led the stakeholders involved to agree on a common goal: the formulation of a national hepatitis B and C elimination strategy, the so-called Swiss Hepatitis Strategy, adopted by Swiss Hepatitis and developed towards the goal of bringing the incidence of new infections, secondary diseases and mortality close to zero by 2030 [27].

By contrast, the FOPH considered the development of an elimination strategy unnecessary [28]. Its main arguments were that risk groups such as drug users should be kept in focus, as most transmissions occur in this group. In fact, drug use makes up 60% of all reported transmissions, but in recent years the majority of infections reported an unknown transmission route [29]. The FOPH further argued that hepatitis C was already covered in the national addiction strategy. In fact, HCV in drug users as an especially affected group is mentioned in the addiction strategy, but only in a single sentence [30].

#### Lifting the limitation

Together with the Swiss Hepatitis C Association (SHCA), a newly founded patient organization, Swiss Hepatitis, campaigned to lift the limitation. The organization considered this crucial to enable access to treatment for all chronically ill patients. International guidelines calling for the treatment of all these patients supported this assessment [26]. In order to provide access to treatment despite the limitations, Swiss Hepatitis facilitated treatment with licensed Indian generics via a buyers' club [31, 32]. This increased the pressure on all actors and brought the FOPH and the pharmaceutical companies back into the negotiations [33]. After the pharmaceutical companies halved the introductory price, the drugs were finally approved for all [34]. This also proved that the limitation had originally been motivated purely by financial reasons, not, as officially stated, for medical considerations regarding the need for treatment.

#### Screening of certain population groups

The majority of researchers and hepatitis experts agreed that those affected should receive treatment at an early stage to prevent consequential damage and additional costs. However, since many infected people are unaware due to the nonspecific symptoms [35], they would have to be specifically “sought out” for treatment. Swiss Hepatitis therefore proposed targeted testing of certain population groups to identify those who were unknowingly affected by HCV. The focus here was on people born between 1950 and 1985, because this group, as an evaluation of the reporting system showed, accounts for two thirds of the positive reported cases [36].

The FOPH, by contrast, saw no urgent need to search specifically for infected persons because, in their view, HCV infections were not a problem if there were no pronounced liver symptoms, and the effort to find the affected persons was disproportionate to the benefit. An FOPH representative stated:

*A huge discussion has developed between us and the professional world as to whether asymptomatic hepatitis C is worth treating or not. If someone is merely infected, is only the carrier, should you really treat them or not? And that automatically leads to the question: do you have to look for them or not? So far, there is no rigorously robust study showing that.*

In the discussion regarding public health measures, the FOPH repeatedly retreated to the position that the primary aim was to prevent new infections. Swiss Hepatitis, on the other hand, insisted that its concerns regarding HCV lay less in new infections than in secondary diseases and the high mortality of those already infected. The FOPH however, pointed to the low and falling numbers of new infections and the stagnating mortality rates as reasons for not taking additional measures against HCV and not aiming for a national strategy, as in their view there was no actual problem [28].

A physician and researcher assessed the situation differently:

*We could link the databases showing that there is a 50% underreporting in the death certificates. […] We’ve been providing these data continuously to the Federal Office. Ironically, the only study which got picked up by the Federal Office years ago was the mortality study showing that the mortality is not increasing. That a little bit killed the discussion. […] The data was quite interesting because for the first time we knew exactly how many people were dying in Switzerland [of hepatitis C]. […] But the problem is that this data was used by the Federal Office to justify their lack of action. […] The bad aspect of this data was the mortality due to hepatitis C was shown to be five times as high [as] the mortality due to HIV. So, ok, it’s not increasing but it’s still five times higher [than HIV]!*

The FOPH rejected the idea of a screening. The authority assessed the problem “as not urgent from an epidemiological, medical and economic point of view” [28]. In this view, people with HCV but without symptoms require no therapy as, despite their infection, they could still be considered healthy in this state. This fundamentally different view represented the main conflict between the FOPH and most hepatitis specialists.

#### Integration into the national HIV programme and the HIV structures

As a measure to ensure sustainable funding (so far, most activities had been made possible by volunteer work and pharmaceutical/foundation funding), the integration of viral hepatitis into the national HIV programme was proposed: instead of an independent, isolated programme for viral hepatitis, Swiss Hepatitis called for existing structures to be used. This made the idea of an official elimination strategy for hepatitis more feasible. The proposal also met the existing values of decision-makers in the health sector, in that this solution was as efficient and cost-effective as possible. Therefore, as far as “anticipating future constraints” [11] were concerned, resistance from politicians or the public because of budgeting or general acceptance was unlikely. The FOPH, however, dismissed the idea in a letter that was addressed to Swiss Hepatitis and several medical associations.

### Political stream: the visible actors

As a result of high drug prices, HCV was conspicuous in the media. The topic also gained more visibility since WHO included hepatitis in the global health sector strategy in a resolution in 2016 [6] (Fig. 1).

#### FOPH and the Federal Council

As the FOPH is rigidly hierarchical, the top cadre exerts considerable influence on its stance. Since key directional positions in the Federal Council and the FOPH have remained unchanged over many years, there was a great deal of continuity of policies. But a crucial change occurred in the Federal Commission for Issues relating to Sexually Transmitted Infections (CFIST) that advises the Federal Council and the administration on the prevention and control of HIV and other sexually transmitted infections: in 2017, an epidemiologist and public health specialist with a broad network among experts took over the presidency. This change was decisive for the subsequent breakthrough of a core concern of Swiss Hepatitis: the new chair advocated that viral hepatitis be integrated into the roadmap for HIV elimination drawn up by the CFIST. This roadmap forms the basis for a national programme for blood-borne and sexually transmitted infectious diseases [37]. Thus, an idea that had been outlined by experts in the policy stream was taken up by an influential actor.

#### Civil society

With the introduction of the new drugs and the FOPH-imposed limitation of their use, civil society—with Swiss Hepatitis at the forefront—weighed in on HCV for the first time on a larger scale. They actively promoted the founding of SHCV in 2017, which for the first time represented patients’ concerns regarding HCV more broadly at the national level. Together, the SHCV and Swiss Hepatitis targeted media attention to raise awareness of the disease, to promote understanding of and solidarity with those affected and to call for lifting of the limitation. Various high-profile activities ensured that HCV retained media attention to keep up the pressure on the FOPH and persuade the pharmaceutical companies to make price concessions [33]. It was important that the disease be given a face by the representatives of SHCV and that they took up a public stance [38]. Numerous media reports about the price debate and patients' fates also shaped the public mood and opinion in favour of HCV sufferers [31, 39–41].

#### International

An important step to increase the visibility of the disease burden due to viral hepatitis was taken in 2010, when the World Health Assembly adopted a resolution “recognizing viral hepatitis as a global public health problem” and calling for a more robust, comprehensive response to the global epidemic [42]. At the 67th World Health Assembly in 2014, WHO referred to hepatitis as an “emerging epidemic”, emphasized what a burden the disease represents for public health, comparable to that of HIV and tuberculosis, and urged its Member States to develop national strategies to combat viral hepatitis [7]. Switzerland as a WHO Member State was among the countries that signed the resolution, thereby making a declaration of intent to develop appropriate measures. In 2016, the 195 WHO Member States at the World Health Assembly finally adopted the Global Health Sector Strategy on Viral Hepatitis 2016–2021, marking a major success in the fight against viral hepatitis [43].

### Policy entrepreneur

The role of the policy entrepreneur was taken on by Swiss Hepatitis, an association which consists of physicians, experts in the field of hepatitis and patients. Until the arrival of the DAA drugs, the association had mainly acted as an advisory and expert organization, but the drug limit imposed by the FOPH politicized several members and led to a new orientation of the association's activities. To achieve the elimination goals within the framework of the hepatitis strategy, the members volunteered more than 3000 hours. In addition, the financial support of pharmaceutical companies enabled a broader scope of action, especially with regard practical projects and to implementing research results (Fig. 1).

#### Dramatization of the problem

To frame the debate and to make the silent disease more tangible, Swiss Hepatitis drew two comparisons: “in Switzerland today five times more people die from the consequences of hepatitis C than from HIV” [44] and “200 people die every year from the consequences of hepatitis C—the same number as from traffic accidents” [45]. The comparison with HIV was intended to illustrate the disproportional financial resources made available by the FOPH for HIV prevention: Sfr. 2 million was spent annually on the national HIV prevention campaign “Love Life” [46] alone, compared to a mere Sfr. 300,000 to combat HCV [47].

#### “Softening-up” process

Swiss Hepatitis initiated and coordinated various studies and publications, whose results appeared in specialist journals. There, they provided information on the new treatment options and current progress in successful treatment, repeatedly drew attention to the poor care situation of HCV-infected individuals and pointed out the large gaps in care [8, 35, 48–51]. Swiss Hepatitis was also frequently present in the mass media, especially in connection with the limited approval of new drugs.

At a meeting in 2015 with the health minister, Swiss Hepatitis proposed a public–private partnership (PPP) to combat the hepatitis epidemic. This prompted the FOPH for the first time to commission a situation analysis [17] on the epidemiology of viral hepatitis in Switzerland, to provide a basis for decision-making regarding the PPP proposal.

From 2015 onwards, to establish a basis for their demands, Swiss Hepatitis also regularly contacted public health policy-makers and provided them with the latest scientific findings. In 2017, they presented their concerns to the public health policy-makers in a meeting of the Council of States Social Security and Health Committee (SGK-SR). At this meeting, Swiss Hepatitis’ request for the FOPH to provide financial support for the Swiss Hepatitis Strategy with the goal of eliminating viral hepatitis fell on receptive ears across party lines. At the end of 2017, in parallel with these discussions, Swiss Hepatitis also presented their concerns to the secretary general of the conference of cantonal health directors, who gave ideological support for the Swiss Hepatitis Strategy and the associated goals and measures [52].

#### Coupling and policy window

With the activities described above, Swiss Hepatitis had prepared the ground: the public had been sensitized through media work and, above all, the important public health policy-makers in the health commissions had been convinced of the necessity of political measures.

With the expiry of the national sexual health programme in 2021, a “natural” policy window presented itself. The prelude to its opening was an interpellation in the Council of States concerning the resources used to combat hepatitis C [47]. The paltry Sfr. 300,000 per year which the Federal Council presented in its response again highlighted the low priority given to combating hepatitis at the federal level and prompted the decisive initiative by the Council of States in summer 2019, when a motion called for the integration of viral hepatitis into the next national HIV programme [53]. This motion was adopted in both chambers of parliament on the recommendation of the Federal Council, which for the first time put hepatitis C prominently on the national political agenda.

As mentioned in the section on the political stream, the Federal Commission for Issues relating to Sexually Transmitted Infections (FCIST) drew up a roadmap for HIV elimination at the same time. The roadmap represents the basis for the development of the 2022–2026 national HIV follow-up programme [37], opening an opportunity to integrate the hepatitis C issue into a national programme on an equal footing with HIV. Swiss Hepatitis’s proposal to integrate viral hepatitis into this roadmap was positively received by the FCIST, and the roadmap was finalized in 2019. This fulfils an important demand of Swiss Hepatitis: to create synergies and access to the federal budget using HIV structures to combat hepatitis C in Switzerland.

## Discussion

This policy analysis used the multiple streams framework described by Kingdon [11] to investigate how the issue of hepatitis C, neglected for decades, came onto the agenda, and which actors and factors were influential in this process.

While the problem stream, among other things, highlighted the large number of undetected HCV infections as a problem that demanded action, in the policy stream, the policy community elaborated proposals on how the goal of hepatitis elimination could be achieved. This vision of eliminating hepatitis represented a common thread, which shaped the individual ideas and measures. The policy entrepreneur played a crucial role in “softening up” the audience: first, via mass media, the broader public was alerted to HCV; next a specialized audience was addressed via specialist publications; and finally, using classic lobbying, health policy-makers were targeted. Thus, measures that were developed in the policy stream convinced visible participants from the political stream that the issue of HCV was important and that the ideas (commitment to elimination, integration of HCV into HIV structures) were feasible and thus warranted acceptance. With the policy window opening up in the political stream through a relaunch of the HIV programme, the policy entrepreneur reacted immediately and introduced an idea already finding acceptance—integrating HCV into HIV structures. Swiss Hepatitis as a policy entrepreneur thus united the three streams, crucial for putting an issue on the agenda.

It was these combined measures and actions of a closely linked hepatitis network that finally helped the cause to achieve a breakthrough. Between 2013 and 2018, the pharmaceutical industry, but also other actors, had tried to persuade politicians to act with individual proposals in parliament—but without success. The fact that politicians were finally persuaded, in addition to the developments described in the case study, was possibly also due to the fact that the estimated number of people affected by HCV had been corrected downwards from 80,000 to 40,000 in a new study, which meant that the treatment costs no longer seemed as imposing as the initial forecasts when the DAAs entered the market.

The debate about HCV highlights the different values and interests of the actors involved: physicians treating patients at an advanced stage of the disease, sometimes including permanent liver damage; authorities calculating cost–benefit and interpreting new study results accordingly; pharmaceutical companies intent on generating as much profit from new products as quickly as possible; and HCV patients suffering from barely measurable but severely limiting symptoms, who want to be treated and benefit from new therapies at all costs.

Thus, this paper represents a typical case study for health policy by showing where the most common lines of conflict in healthcare are. The problems most commonly encountered in healthcare are “the big three”: cost, access, quality [11]. In the present case study, too, they played a central role in the conflict: it was the high cost of the new therapy that prompted the FOPH to introduce a limitation, so that initially only people with advanced liver damage were given access to treatment with the new DAAs. This measure, initiated for economic reasons, was medically justified, whereby this justification was based on outdated data in medical and epidemiological questions about hepatitis C, which suddenly took on much greater significance with the medically quasi-unrestricted application possibilities of the DAAs. The high medical quality of DAAs could not be fully exploited due to the cost-related limited access to therapy.

This study is a case study from a high-income country. The developments and measures described here can only be transferred to a limited extent to middle- and low-income countries. For example, in countries where civil society is weak, it is not possible to build up a network like Swiss Hepatitis. In addition, in many places there is a lack of resources for the various initiatives and projects, which in this case were made possible by the financial support of foundations and pharmaceutical companies.

In the future, Swiss Hepatitis will be called upon to participate in developing the HIV and HCV programme and will continue to exert influence through various channels. It must ensure that the members of the States Council who called for the integration of hepatitis into the HIV programme are kept up to date regarding the progress of the follow-up programme, so that they can intervene politically if necessary. If the implementation does not go as planned, the problem stream will open again. The extent to which such an implementation succeeds should be the subject of future research.

## Conclusion

The case of HCV in Switzerland shows that health policy development can be made possible thanks to a policy entrepreneur who, with years of commitment at different levels, brings together the three streams—problem, policy and political—and exploits the window of opportunity at the right time. To be successful, the policy entrepreneur must identify the indicators that map the problem, network and convince decision-makers and the general public and, importantly, be prepared for and recognize policy window opportunities in order to seize them.

But the case also demonstrates that evidence alone is not enough to bring about health policy changes. It therefore takes a policy entrepreneur to “translate” from science language to everyday language, since science and policy systems are often completely decoupled. As Kingdon points out, “[T]he data do not speak for themselves. Interpretations of the data transform them from statements of conditions to statements of policy problems” [11]. Interpreting and communicating the facts is therefore crucial in determining whether or not something is considered a problem—or merely a condition that does not need to be changed. Clear and catchy messages are important to make the often technical facts understandable and to appeal to people emotionally. In addition, targeted political lobbying is a key task of the policy entrepreneur. The present case is a good example of the fact that with an active policy entrepreneur, a good network and tenacious perseverance, policy change can be achieved—even in health policy with its divergent interests.

## Data Availability

The datasets used and/or analysed during the current study are available from the corresponding author on reasonable request.

## Abbreviations

CFIST: Federal Commission for Issues relating to Sexually Transmitted Infections
DAA: Direct-acting antivirals
FOPH: Federal Office of Public Health
HCV: Hepatitis C
HIV: Human immunodeficiency virus
MSF: Multiple streams framework
